# Supplementary material for: The genome sequence of the Heart and Club moth, Agrotis clavis (Hufnagel, 1766)
Source: Wellcome Open Res. 2023 Oct 12;8:446. [Version 1] doi: 10.12688/wellcomeopenres.20125.1 (PMC11489838; doi:10.12688/wellcomeopenres.20125.1)
Supplement: Peer review history

# Reviewer report: Reviewer response for version 1

**DOI:** 10.21956/wellcomeopenres.22287.r103360

**Published:** 2024-10-18

**Authors:** Pedro Ribeiro

**Affiliations:** 1 Czech Academy of Sciences, České Budějovice, Czech Republic

The author presents a Data Note for the whole-genome, reference level sequencing of *Agrotis clavis *(the Heart and Club moth). Background is concise and, despite including some aspects of the moth's biology and distribution, could include more on the rationale behind the study of this particular moth. 

 Methods, as usual, present all aspects developed by the Sanger, from sample acquisition to assembly of the PacBio HiFi and Illumina Hi-C generated sequences, the two technologies responsible for the generation of this genome.

Are sufficient details of methods and materials provided to allow replication by others?

Yes

Is the rationale for creating the dataset(s) clearly described?

No

Are the datasets clearly presented in a useable and accessible format?

Yes

Are the protocols appropriate and is the work technically sound?

Yes

Reviewer Expertise:

Evolutionary biology, phylogenetics and biogeography of Neotropical butterflies.

I confirm that I have read this submission and believe that I have an appropriate level of expertise to confirm that it is of an acceptable scientific standard.

# Reviewer report: Reviewer response for version 1

**DOI:** 10.21956/wellcomeopenres.22287.r90588

**Published:** 2024-10-08

**Authors:** Sara Goodwin, Shruti Iyer

**Affiliations:** 1 Cold Spring Harbor Laboratory, New York, USA; 2 Cold Spring Harbor Laboratory, Cold Spring Harbor, New York, USA

Review of “The genome sequence of the Heart and Club moth, Agrotis clavis (Hufnagel, 1766)”

 The Darwin Tree of Life Project is an exciting genomic endeavor. This latest manuscript reads as a typical article that is part of this project and is concise, while providing relevant details. I have a few minor suggestions for the authors.

  

1. Purely from an experimental curiosity standpoint, perhaps the authors could elaborate on the following:

“The concentration of the sheared and purified DNA was assessed using a Nanodrop spectrophotometer and Qubit Fluorometer and Qubit dsDNA High Sensitivity Assay kit. Fragment size distribution was evaluated by running the sample on the FemtoPulse system.”

 How much material was obtained on DNA extraction and what was the size distribution like as seen on the femtopulse?

Are sufficient details of methods and materials provided to allow replication by others?

Yes

Is the rationale for creating the dataset(s) clearly described?

Yes

Are the datasets clearly presented in a useable and accessible format?

Yes

Are the protocols appropriate and is the work technically sound?

Yes

Reviewer Expertise:

Sequencing, genomics, non-model organisms.

We confirm that we have read this submission and believe that we have an appropriate level of expertise to confirm that it is of an acceptable scientific standard.
